# New phiocricetomyine rodents (Hystricognathi) from the Jebel Qatrani Formation, Fayum Depression, Egypt

**DOI:** 10.7717/peerj.12074

**Published:** 2021-10-19

**Authors:** Shorouq F. Al-Ashqar, Erik R. Seiffert, Dorien de Vries, Sanaa El-Sayed, Mohamed S. Antar, Hesham M. Sallam

**Affiliations:** 1 Department of Geology, Mansoura University Vertebrate Paleontology Center (MUVP), Faculty of Science, Mansoura University, Mansoura, Egypt; 2 Department of Integrative Anatomical Sciences, Keck School of Medicine of USC, University of Southern California, Los Angeles, CA, USA; 3 Ecosystems and Environment Research Centre, School of Science, Engineering and Environment, University of Salford, Manchester, UK; 4 Geology and Paleontology Department, Nature Conservation Sector, Egyptian Environmental Affairs Agency, Cairo, Egypt; 5 Institute of Global Health and Human Ecology (I-GHHE), School of Sciences and Engineering, American University in Cairo, New Cairo, Egypt

**Keywords:** Paleogene, Rodentia, Oligocene, Eocene, Africa

## Abstract

**Background:**

The rich rodent assemblages from the Eocene–Oligocene deposits of the Jebel Qatrani Formation (Fayum Depression, Egypt) have important implications for our understanding of the origin and paleobiogeography of Hystricognathi, a diverse clade that is now represented by the Afro-Asiatic Hystricidae, New World Caviomorpha, and African Phiomorpha.

**Methods:**

Here we present previously undescribed material of the enigmatic hystricognath clade Phiocricetomyinae, from two stratigraphic levels in the lower sequence of the Jebel Qatrani Formation—a new genus and species (*Qatranimys safroutus*) from the latest Eocene Locality 41 (~34 Ma, the oldest and most productive quarry in the formation) and additional material of *Talahphiomys lavocati* from that species’ type locality, early Oligocene Quarry E (~31–33.2 Ma).

**Results:**

The multiple specimens of *Qatranimys safroutus* from L-41 document almost the entire lower and upper dentition, as well as mandibular fragments and the first cranial remains known for a derived phiocricetomyine. Specimens from Quarry E allow us to expand comparisons with specimens from Libya (late Eocene of Dur at-Talah and early Oligocene of Zallah Oasis) that have been placed in *T. lavocati*, and we show that the Dur at-Talah and Zallah specimens do not pertain to this species. These observations leave the Fayum Quarry E as the only locality where *T. lavocati* occurs.

## Introduction

Hystricognathi is a diverse clade of rodents that is characterized by a mandibular angular process situated lateral to the long axis of the lower incisor, multiserial Hunter-Schreger bands of incisor enamel, and enlarged infraorbital foramina, among other features (Tullberg, 1899; Marivaux et al., 2018). Hystricognaths likely originated in the middle Eocene (Marivaux & Boivin, 2019), and in a short time window diversified and radiated across three continents: Asia, South America, and Afro-Arabia (Marivaux & Boivin, 2019). Each of these epicenters housed a distinctive clade—Hystricidae (Old World porcupines), Caviomorpha (New World hystricognaths), and Phiomorpha (African cane, dassie, and mole rats), respectively (Singleton, Dickman & Stoddart, 2006). The Asian tropics are considered to have been the ancestral homeland for Hystricognathi (Sallam, Seiffert & Simons, 2011; Barbière & Marivaux, 2015) despite the fact that the oldest known fossil occurrences of hystricognaths are from Africa (Sallam et al., 2009; Marivaux et al., 2014). Numerous molecular studies have placed Hystricidae as the sister group of a Caviomorpha-Phiomorpha clade (*e.g*., Huchon et al., 2007; Meredith et al., 2011; Patterson & Upham, 2014; Campbell et al., 2021). Caviomorpha and Phiomorpha are estimated to have split around 39–43 Ma (Sallam & Seiffert, 2016; Patterson & Upham, 2014).

**Figure 1:**
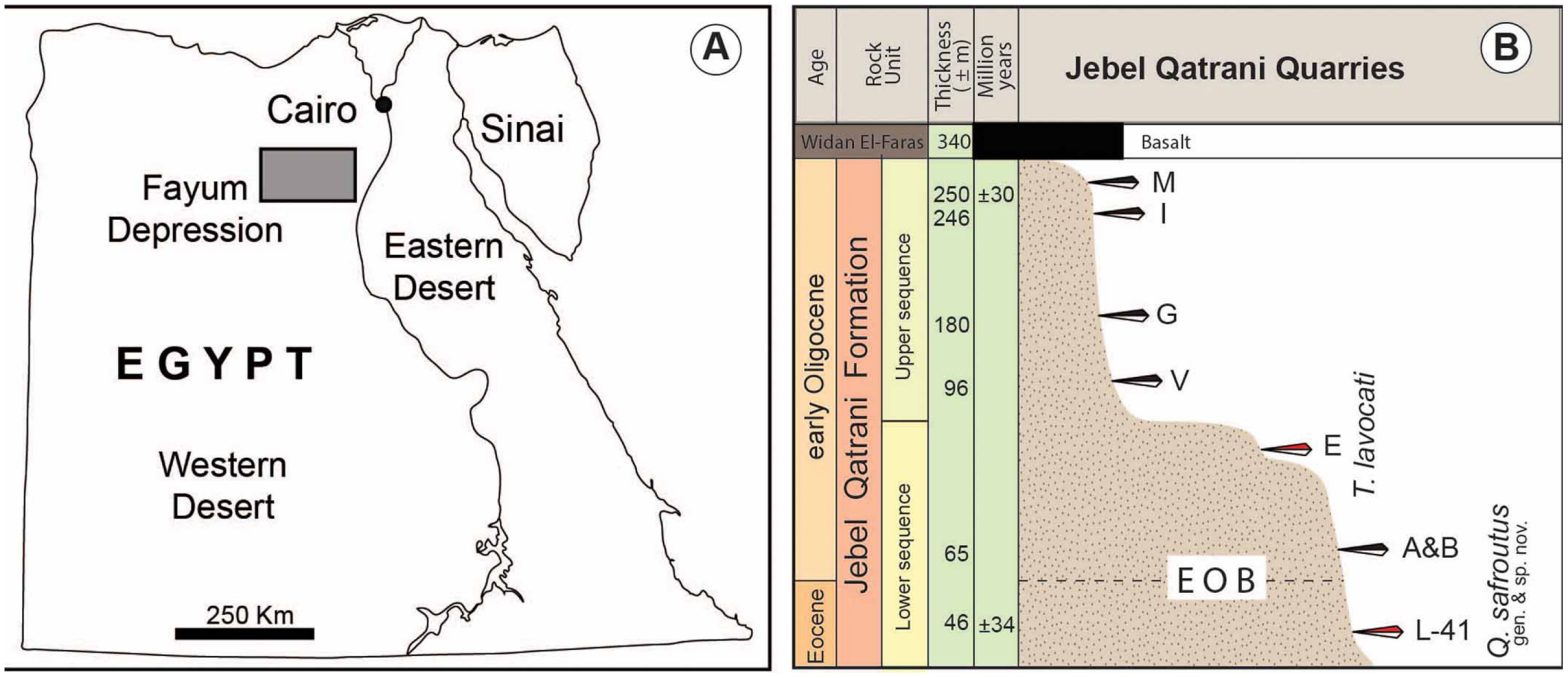
(A) Map of Egypt, the grey rectangle shows the location of the Fayum Depression; (B) stratigraphic column of Jebel Qatrani Formation shows the distribution of vertebrate quarries.

Phiocricetomyinae is an enigmatic Afro-Arabian clade of small hystricognaths with bunodont and simple low-crested cheek teeth whose core members are currently known solely from dental remains. The fossil record of this group is very limited, and its phylogenetic position relative to Phiomorpha is uncertain; phiocricetomyines have variously been placed outside of the Phiomorpha-Caviomorpha clade (Sallam et al., 2009; Sallam, Seiffert & Simons, 2011; Sallam & Seiffert, 2016; Marivaux & Boivin, 2019) or as stem phiomorphs (Sallam & Seiffert, 2016, 2019), suggesting that this group is of key importance for understanding polarities of dental characters near the base of the hystricognath radiation. In 1968, Wood described the first genus and species of this group, a peculiar and highly derived form represented by a mandible with dP_4_-M_2_ that he named *Phiocricetomys minutus*; it remains the youngest known member of Phiocricetomyinae, being from one of the youngest fossil-bearing levels (Quarry I, ~29–30 Ma) (Fig. 1) in the Fayum Depression. Lavocat (1973) later created the subfamily Phiocricetomyinae to contain *P. minutus*. Phylogenetic analyses of early Afro-Arabian Hystricognathi (Holroyd, 1994; Sallam et al., 2009; Sallam, Seiffert & Simons, 2011, 2012; Sallam & Seiffert, 2016, 2019) have since placed another taxon described by Wood (1968)—early Oligocene “*Phiomys*” *lavocati* from the ~31–33.2 Ma Fayum Quarry E (Fig. 1)—with *Phiocricetomys* to the exclusion of other hystricognaths. Published remains of “*P*.” *lavocati* from Quarry E have thus far been limited to dP_4_-M_3_, and published figures of these specimens are highly schematic line drawings.

In her doctoral dissertation, Holroyd (1994) was the first to propose a relationship between “*Phiomys*” *lavocati* and *Phiocricetomys*, wherein she proposed the new genus *Elwynomys* for “*P*.” *lavocati*—a decision based on insights provided by new material from L-41 (see below) and the type locality for “*P*.” *lavocati* (Quarry E) (Fig. 1). However, the name *Elwynomys* was never published in a way that satisfies the criteria of the International Commission on Zoological Nomenclature (ICZN). Jaeger et al. (2010) subsequently erected the generic name *Talahphiomys* for “*P*.” *lavocati*, based on their study of isolated teeth collected from the Idam Unit of the Dur at-Talah escarpment in central Libya that those authors considered to be conspecific with “*P*.” *lavocati*. Using the new material from Quarry E described here, we show that the specimens from Dur at-Talah actually do not belong in the species *lavocati*, but *Talahphiomys* nevertheless has priority as the generic replacement name for “*P*.” *lavocati*. Coster et al. (2012) has since identified isolated lower teeth from the early Oligocene “rodent locality 5” (ZR5) in the Zallah Oasis of Libya as *T. lavocati*, but we are able to demonstrate that the ZR5 specimens also do not belong in that species, and that *T. lavocati* is restricted to the Fayum Quarry E. Extensive new material of an additional new phiocricetomyine genus and species from the latest Eocene Quarry L-41 (herein named *Qatranimys safroutus*, see below) provides the first detailed insights into the craniodental morphology of, and intraspecific variation within, a phiocricetomyine species.

### Fossil localities

The late Eocene–early Oligocene Jebel Qatrani Formation (Seiffert, 2006) that is exposed north-northwest of Birket Qarun in the Fayum Depression, Egypt (Fig. 1), has produced an extensive fossil record of distinctive and diverse clades of terrestrial mammals. The Jebel Qatrani Formation has been interpreted as a primarily fluvial deposit and is characterized by abundant weathering horizons and root traces indicative of a tropical monsoon climate regime (Bown & Kraus, 1988). The formation has been separated into an upper sequence and a lower sequence, with the division between the two sequences marked by a cliff-forming unit called the “Barite Sandstone”. The fossils described here come from the type locality for *T. lavocati* (Quarry E) and the older Quarry L-41 (Fig. 1). Based on the preferred paleomagnetic correlation of Seiffert (2006), L-41 is estimated to be between 33.9–35 Ma, while Quarry E is estimated to be between 31 and 33.2 Ma. L-41 is located approximately 48 m above the base of the Jebel Qatrani Formation and just below a major unconformity that has been identified as the most likely site of near-shore erosion during early Oligocene sea level fall (Seiffert, 2006). L-41 is a well-consolidated deposit that is dominated by clay and post-depositional salt, contrasting with the fine- to medium-grained sandstones of younger Fayum quarries (Bown & Kraus, 1988). L-41 is the richest Paleogene vertebrate site in Africa and preserves hundreds of thousands of fossils such as bats (Gunnell, Simons & Seiffert, 2008), rodents (Sallam, Seiffert & Simons, 2011, 2012; Sallam & Seiffert, 2016), and primates (Simons, 1990, 1997; Simons et al., 2001; Seiffert et al., 2018). Quarry E is located approximately 90 m above the base of the Jebel Qatrani Formation and is composed of unconsolidated gravelly sandstones that were deposited as point bars in large meandering rivers (Simons & Rasmussen, 1990). Quarry E has yielded a great diversity of vertebrate fossils such as rodents (Wood, 1968), birds (Stidham & Smith, 2015), and anthropoids (Simons, 1962; Simons & Kay, 1983).

## Materials & methods

### Taxonomy

The electronic version of this article in Portable Document Format (PDF) will represent a published work according to the International Commission on Zoological Nomenclature (ICZN), and hence the new names contained in the electronic version are effectively published under that Code from the electronic edition alone. This published work and the nomenclatural acts it contains have been registered in ZooBank, the online registration system for the ICZN. The ZooBank Life Sciences Identifiers (LSIDs) can be resolved and the associated information viewed through any standard web browser by appending the LSID to the prefix http://zoobank.org/. The LSID for this publication is urn:lsid:zoobank.org:pub:79E437BD-03EA-42BD-B341-D77EF2AC37F7. The online version of this work is archived and available from the following digital repositories: PeerJ, PubMed Central SCIE and CLOCKSS.

### Dental cusp and crest nomenclature

**Figure 2:**
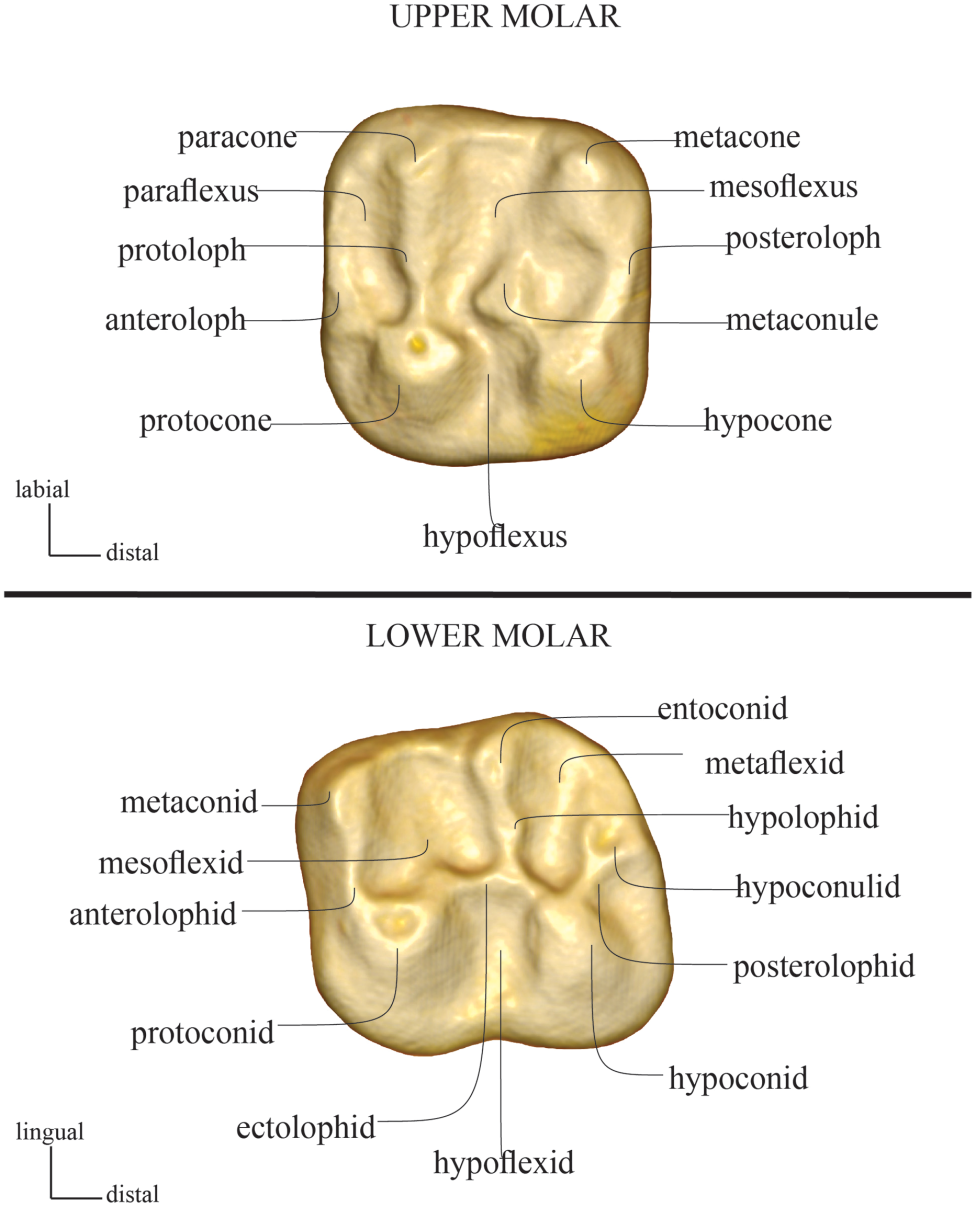
Dental cusp and crest nomenclature, following Marivaux & Boivin (2019).

Terminology follows Marivaux & Boivin (2019) (Fig. 2). Teeth are referred to as I, P, and M (for incisors, premolars, and molars, respectively), with upper and lower teeth designated by superscript and subscript numbers (respectively) (*e.g*., the second lower molar is referred to as M_2_).

CT scanning and rendering: μ-CT scans of the original fossils of *Talahphiomys lavocati* and *Qatranimys safroutus*, and of casts of the original fossils of the Dur at-Talah specimens of “*T. lavocati*”, were collected at either the Duke University Shared Materials Instrumentation Facility or the USC Molecular Imaging Center using a Nikon XT H 225 ST micro-CT scanner. Three-dimensional surface models were constructed using ImageJ and Avizo v. 8 and saved in Stanford “ply” format. Additional surface model manipulation and measurements were conducted in Avizo and MeshLab. Digital models of all specimens scanned as part of this study are available on MorphoSource (see Data S1)

## Results

**Systematic Paleontology**

**Class** Mammalia Linnaeus, 1758

**Order** Rodentia Bowdich, 1821

**Infraorder** Hystricognathi Tullberg, 1899

**Parvorder** Phiomorpha Lavocat, 1967

**Family** *Incertae sedis*

**Subfamily** Phiocricetomyinae Lavocat, 1973

**Figure 3:**
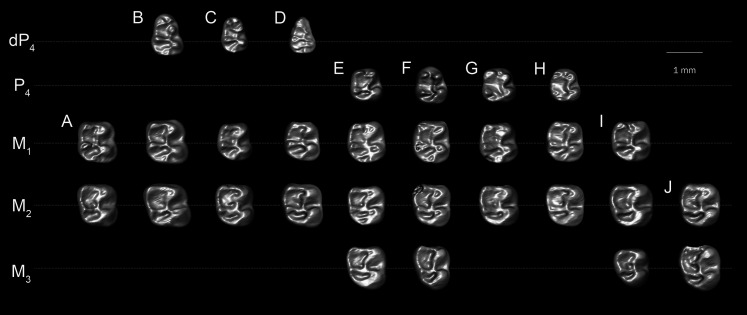
Lower molars of *T. lavocati* from Quarry E (A–B) and *Q. safroutus* from Quarry L-41 (C–J). *T. lavocati*: (A) DPC 8181, left M_1–2_ (reversed); (B) DPC 5057, right dp_4_-M_2_; *Q. safroutus*: (C) DPC 17947, left with dP_4_-M_2_ (reversed); (D) DPC 14187, right dP_4_-M_2_; (E) DPC 10710, left P_4_-M_3_ (reversed); (F) CGM 83743, holotype, right P_4_-M_3_; (G) DPC 14393, left P_4_-M_2_ (reversed); (H) DPC 8825, right P_4_ and M_1-2_; (I) DPC 14056 left M_1-3_; (J) DPC 21818, left M_2-3_ (reversed).

*Talahphiomys lavocati*, Wood, 1968 (Fig. 3; Figs. 5A–5E in Wood, 1968)

***Synonymy***

*Elwynomys lavocati* (in part) in Holroyd (1994, specimen in lowest frame of her Fig. 4.10)

*non Elwynomys lavocati* in Lewis and Simons (2001)

*non Talahphiomys lavocati* in Jaeger et al. (2010, Figs. 6K–6V)

*non Talahphiomys lavocati* in Coster et al. (2012, Fig. 4L)

*non Talahphiomys lavocati* in Marivaux et al. (2014, Figs. 6E–6F)

***Revised diagnosis***

**Figure 4:**
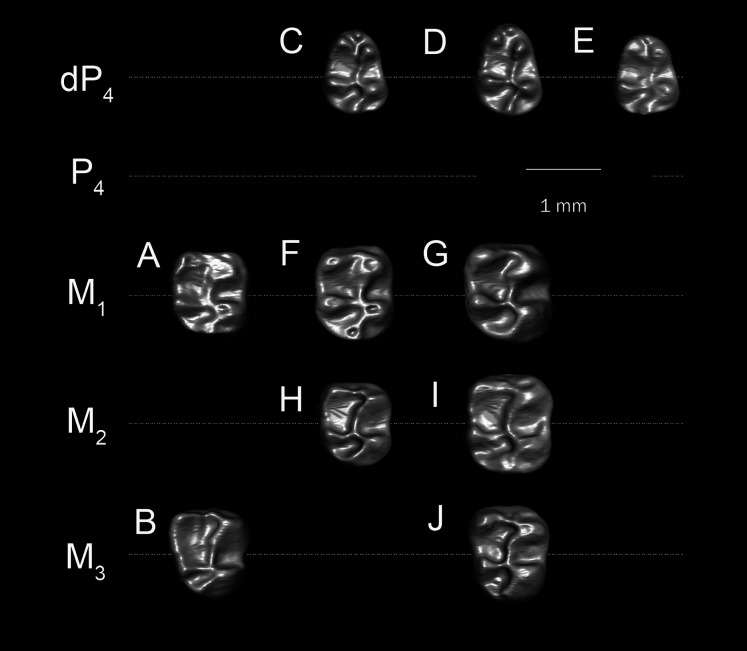
Lower molars of “*T. libycus*” (A–B) and “*T. lavocati*” (C–J) from Dur at-Talah DT-LOC-2, Libya. (A) DT-1-024, left M_1_ (reversed); (B) DT-1-025, left M_3_ (reversed); (C) DT-2-016, left dP_4_ (reversed); (D) DT-2-020, left dP_4_ (reversed); (E) DT-2-unnumbered, left dP_4_ (reversed); (F) DT-2-021, right M_1_? (G) DT-2-022, right M_1_? (H) DT-2-017, left M_2_? (reversed); (I) DT-2-018, left M_2_ (reversed); (J) DT-2-019, left M_3_ (reversed).

**Figure 5:**
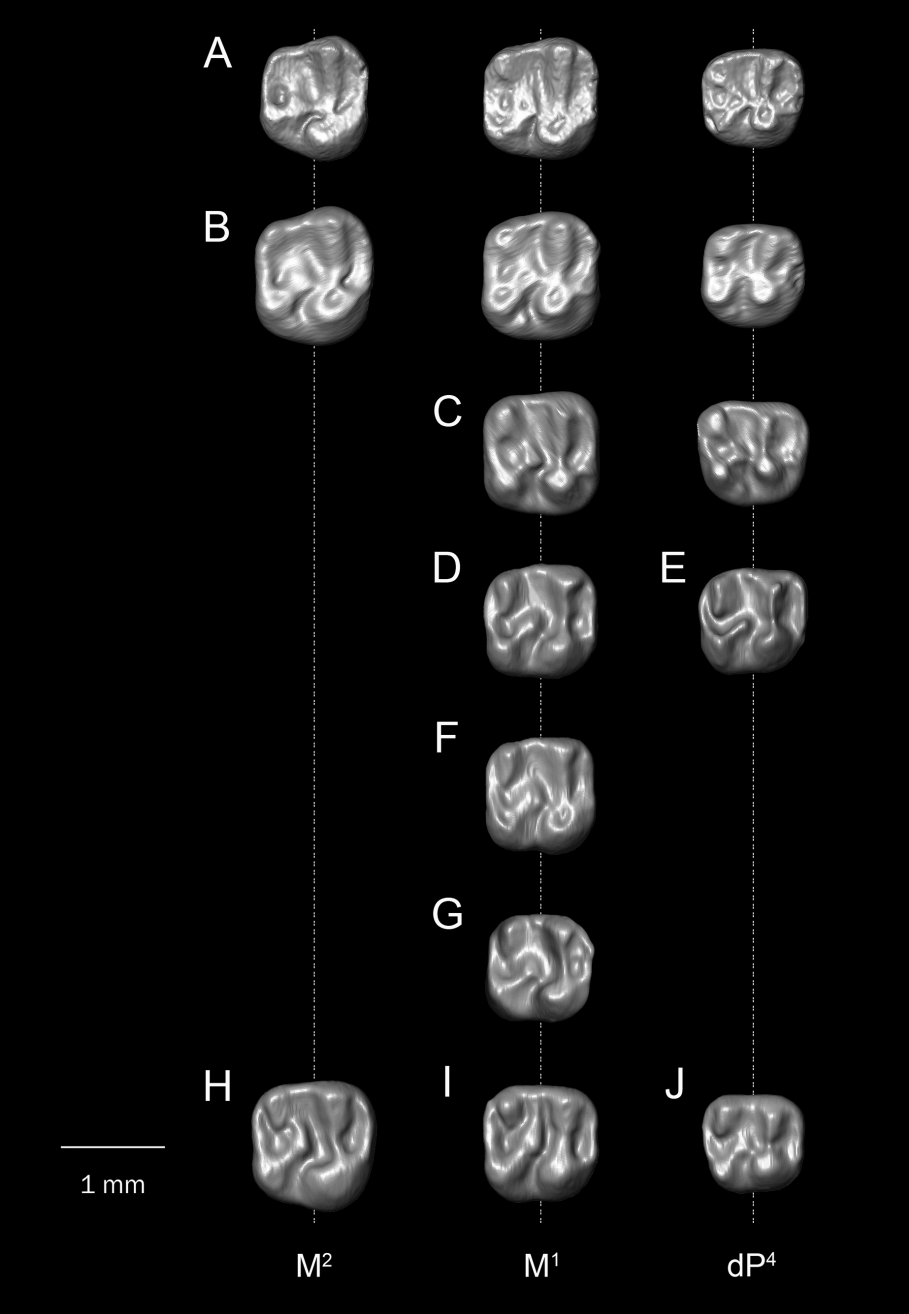
Comparison among upper molars. (A and B) *Q. safroutus*, DPC 16815, left dP^4^-M^2^ (reversed); (B) DPC 10300, left dP^4^-M^2^ (reversed); (C) *T. lavocati* from Quarry E, DPC 4275, left dp^4^-M^1^ (reversed); (D–H) “*T. lavocati*” from Dur at-Talah DT-LOC-2, Libya, (D) DT-2-014, right M^1^; (E) DT-2-015, left dP^4^ (reversed); (F) DT-2-unnumbered, right M^1^; (G) DT-2-unnumbered, left M^1^ (reversed); “*T. libycus*” from Dur at-Talah DT-LOC-1, Libya, (H) DT-1-021, right M^2^; (I) DT-1-022, left M^1^ (reversed); (J) DT-1-023, right dP^4^.

**Figure 6:**
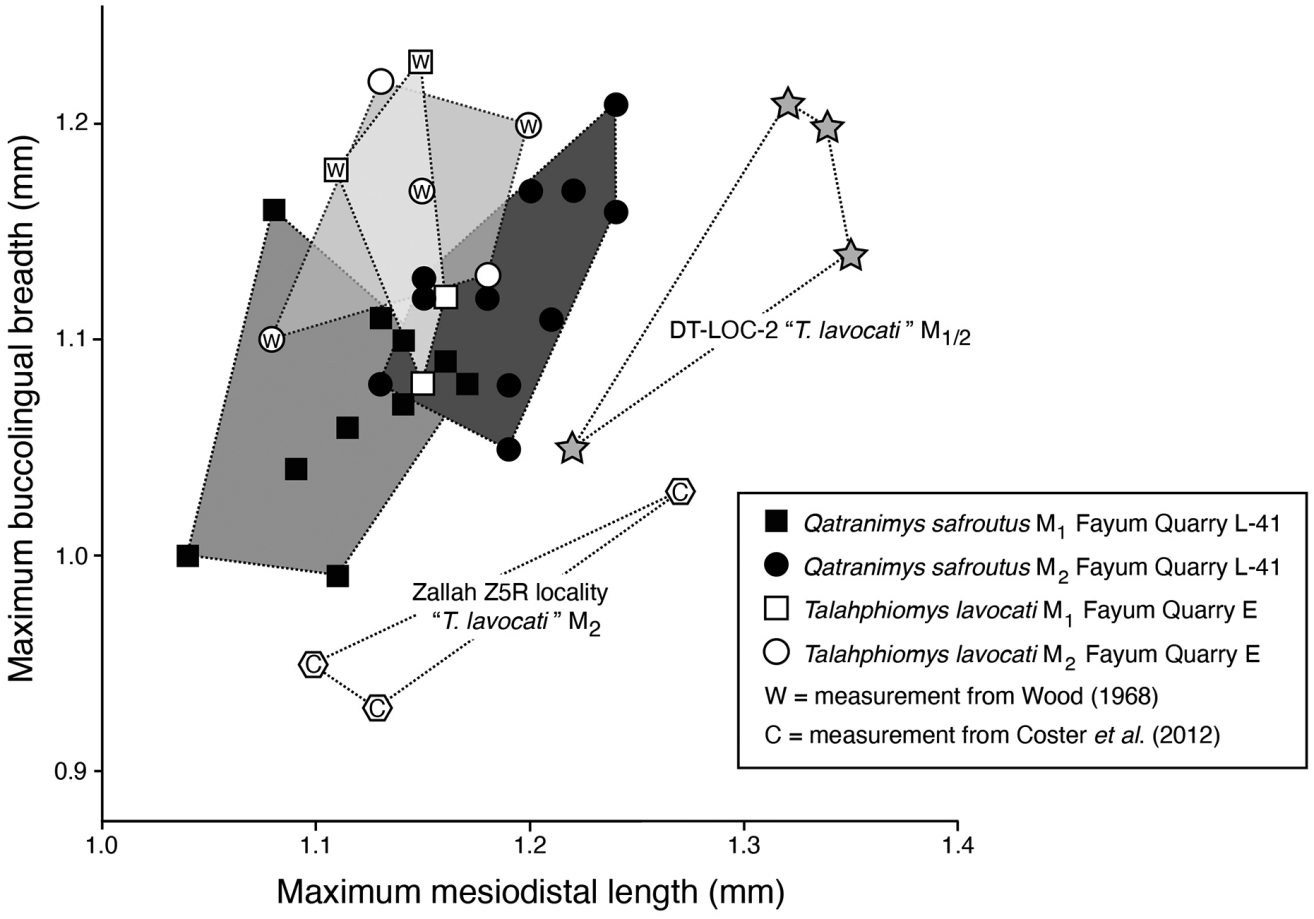
Bivariate plot of maximum mesiodistal length (*x*-axis) *vs* maximum buccolingual breadth (*y-*axis) of M^1^ and M^2^ in *Talahphiomys lavocati* from Fayum Quarry E, “*T. lavocati*” from Dur at-Talah DT-LOC-2 and Zallah ZR5, and *Qatranimys safroutus* from Fayum Quarry L-41.

*T. lavocati* (Figs. 3–5) differs from *Talahphiomys libycus* from Dur at-Talah DT-LOC-1 in having a relatively long dP^4^ with a larger paraflexus. The mesoflexus bears a weak metaloph connecting to the metaconule. *T. lavocati* differs also in lacking a mesostyle, mesolophule, and isolated metaloph on M^1^ (the latter being submerged into the posteroloph in *T. lavocati*). *T. lavocati* differs from *Qatranimys safroutus* (new genus and species, see below) (Figs. 3–5) in having inflation of the enamel surrounding the base of the protoconid and to a lesser extent the hypoconid of M_1–2_, forming an incipient labial cingulid; broad overlap in the size and proportions of M_1_ relative to M_2_ (see bivariate plot in Fig. 6); a lingually open M_2_ metaflexid, with no tall connection of the posterolophid to the entoconid; mesial and lingual margins of the mesiolingual corner of M_1–2_ that form roughly a 90 degree angle, rather than a relatively obtuse angle; a relatively long dP^4^ with a labial margin longer than the lingual margin, a relatively capacious paraflexus, and a relatively lingually placed paracone; and an M^1^ that is relatively quadrate in occlusal view. Differs from the “*T. lavocati*” specimens from Dur at-Talah Locality (DT-LOC-2) (Figs. 4–5) in having inflation of the enamel surrounding the base of the protoconid and to a lesser extent the hypoconid of M_1–2_, forming an incipient labial cingulid; smaller M_1–2_ with different proportions (being mesiodistally longer than buccolingually broad; see bivariate plot in Fig. 6); a more distally placed dP_4_ protoconid, and a deep sulcus between that cusp and the adjacent metaconid and anteroconid; a relatively well developed posterior arm of the metaconid and anterior arm of the entoconid on M_1–2_, closing the mesoflexid lingually; relatively deep indentations on the crown wall of M^1^ mesial to the protocone, forming an incipient anterocingulum; dP^4^ that is relatively trapezoidal in occlusal outline, with a shorter anterior arm of the hypocone, larger paraflexus, and incipient connection of the metaloph with the metacone. Differs from the “*T. lavocati*” specimens from Locality ZR5 (Zallah Oasis) in having M_1–2_ that are relatively broad compared to mesiodistal length (see bivariate plot in Fig. 6), and in having a relatively well-developed posterior arm of the metaconid and anterior arm of the entoconid on M_2_, closing off the mesoflexid lingually. *T. lavocati* differs from *Phiocricetomys minutus* (Wood, 1968; Fig. 16) in retaining M_3_ and in having quadrangular (rather than mesiodistally elongate) lower dP_4_-M_2_ with less bulbous cusps and well-developed metalophulid I, ectolophid and posterolophid crests, as well as lingual closure of the trigonids through connection of the posterior arm of the metaconid and anterior arm of the entoconid.

***Holotype***

CGM 26903, right mandible with dP_4_-M_3_ (early Oligocene Quarry E, Jebel Qatrani Formation, Egypt).

***Revised hypodigm***

YPM 18011, left mandible with dP_4_-M_1_; YPM 18057, left mandible with dP_4_-M_2_; DPC 4275, left maxilla with dp^4^-M^1^; DPC 5057, right mandible with dp_4_-M_2_ and incisor; DPC 8181, left mandible with M_1–2_ and incisor (See Table 1 & Data S1).

**Table 1:** Mesiodistal length and buccolingual width of teeth (in millimeters) in the hypodigm of *Talahphiomys lavocati* from Quarry E of the Jebel Qatrani Formation.

Specimen	Side	Upper teeth									
		dP^3^		dP^4^		M^1^		M^2^		M^3^	
		Length	Width	Length	Width	Length	Width	Length	Width	Length	Width
DPC 4275	left	–	–	1.07	1.03	1.12	1.20	–	–	–	–
		Lower teeth									
		dP_4_		P_4_		M_1_		M_2_		M_3_	
		Length	Width	Length	Width	Length	Width	Length	Width	Length	Width
CGM 26903	right					1.08	1.10	1.15	1.16	1.10	1.03
DPC 5057	right	1.10	0.81	–	–	1.15	1.08	1.13	1.22	–	–
DPC 8181	left	–	–	–	–	1.16	1.12	1.18	1.13	–	–
YPM 18011	left	1.12	0.91			1.15	1.17				
YPM 18057	left	1.32	0.87			1.20	1.20	1.20	1.10		
No.		3		0		5		4		1	
Mean		1.18	0.86	–	–	1.148	1.12	1.165	1.15	−1.10	−1.03

***Description of new specimens***

**Figure 7:**
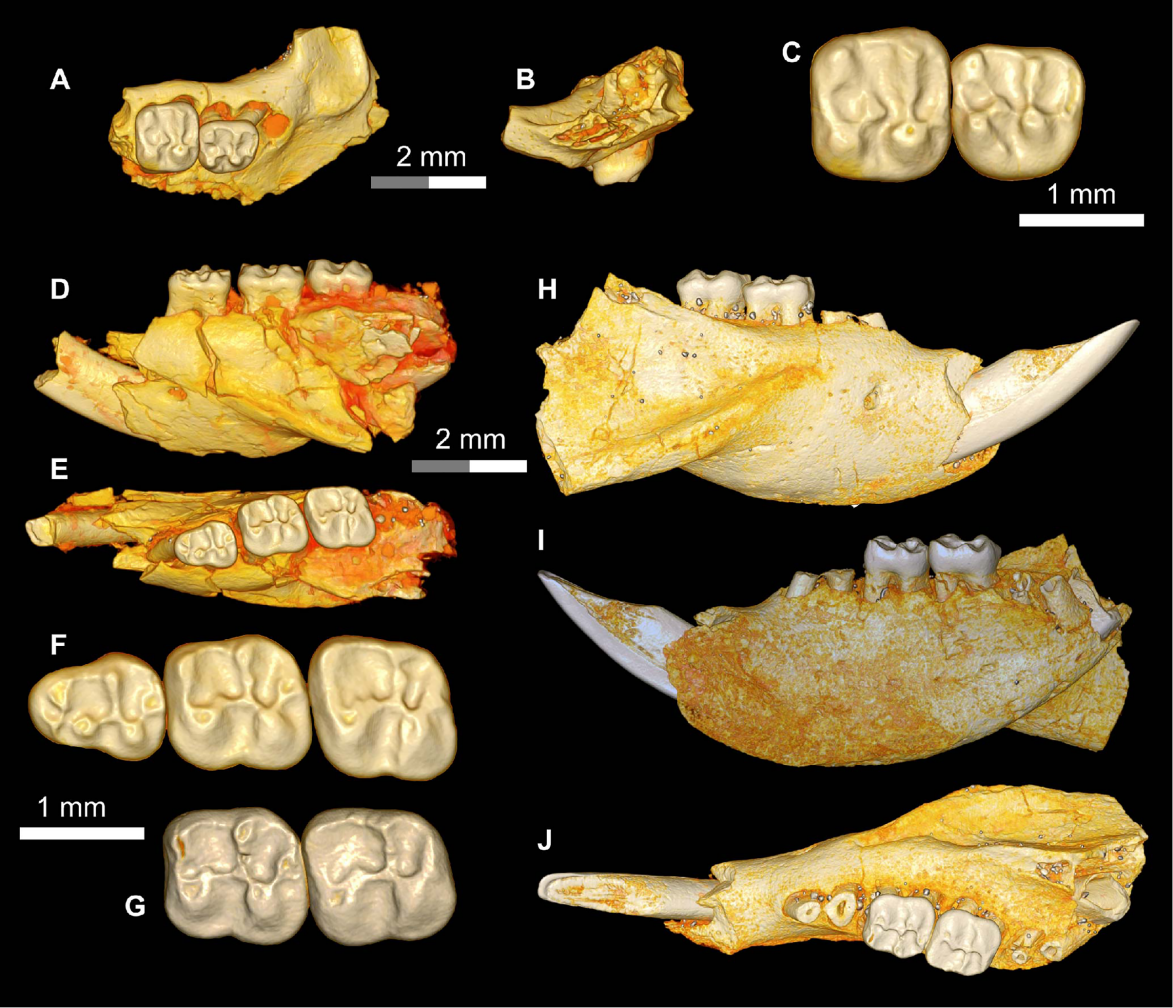
Additional remains of *Talahphiomys lavocati* from Quarry E. (A–C) DPC 4275, partial maxilla with P^4^-M^1^, in (A) occlusal and (B) anterior views, and (C) close-up of crowns of P^4^-M^1^; (D–F), DPC 5057, almost complete right mandible with lower incisor and dP_4_-M_2_ in (D) lateral and (E) occlusal views, and (F) close-up view of occlusal surface of dP_4_-M_2_; (G–J) DPC 8181, almost complete left mandible with lower incisor and M_1-2_, (G) close-up view of reversed M_1-2_ and (H) lateral, (I) medial, and (J) reversed occlusal views. Each of the grey and/or white division in the scale bar represents 1 mm.

The mandible (Fig. 7) is fully hystricognathous, owing to the placement of the angular process lateral to the long axis of the incisor, leaving a distinct groove between the angular process and the incisor alveolus. On the lateral aspect of the mandible, the mental foramen is relatively small, roughly oval in shape and situated directly under the mesial part of the dP_4_ (Fig. 7H). The masseteric fossa is defined dorsally by a weakly-developed dorsal masseteric ridge that fades below the anterior part of M_1_. The ventral masseteric ridge is well developed and originates below the anterior part of M_1_ and continues posteroventrally towards the angular process. The dorsal and ventral ridges meet inferior to the distal part of dP_4_. The posterior portion of the ascending ramus is not preserved in any of the specimens, so the morphology of the coronoid, condylar and angular processes are not known; however, the coronoid process seems to be higher than the tooth row, rising lateral to the third molar and leaving a deep fossa. On the medial surface of the mandible, the angular process originates ventral to M_3_ (Fig. 7I). There are some nutrient foramina scattered on the *corpus*. The outline of the ventral surface of the *corpus* is convex, with the deepest point being beneath the distal portion of the diastema. The diastema is slightly deeper than the alveolar plane and makes up about one-third of the tooth row. The mandibular symphysis is unfused and extends posteriorly to the position below the dP_4_. The symphysis has an anterior part that is broader than its posterior portion (Fig. 7I).

DPC 8181 shows that the lower incisor’s alveolus extends posteriorly to end behind the tooth row. The tooth is covered anteriorly by smooth enamel that extends to the labial and lingual surfaces. On the labial side of the incisor, the enamel covers about one-third, but only a quarter of the medial side. The pulp cavity is exposed as a small slit situated at the middle of the incisor.

A well preserved dP_4_ is implanted in DPC 5057 (Fig. 7F). Its trigonid is narrower than the talonid, and the crown is longer mesiodistally than buccolingually broad. The crown displays five major bulbous cusps (metaconid, protoconid, entoconid, hypoconid and hypoconulid) that are more or less equal in size. On the mesial portion of the crown, the protoconid is positioned distolabially with respect to the metaconid, leaving a somewhat broad mesial shelf; within this shelf there is a short low cristid that is protruding from an incipient anteroconid to reach the metaconid. The anteroconid is placed mesial to the protoconid. The crown lacks a metalophulid I, and there is a notch separating the protoconid from the metaconid and anteroconid that continues into the central basin. The entoconid is placed mesial to the hypoconid, and they are linked by the hypolophid and a well-developed anterior arm of hypoconid. The junction of these two cristids is also the point of connection of a relatively short ectolophid. There is no trace of a mesostylid. On the very distal portion of the crown at its midpoint, there is a well-developed hypoconulid. This hypoconulid connects to the hypoconid *via* a short posterolophid but does not reach the entoconid. The posterior basin is open lingually.

The M_1_ (Figs. 7F–7G) is roughly square in shape, and has five distinct major cusps (protoconid, metaconid, hypoconid, entoconid, and a well-developed hypoconulid). The labial cusps are larger in size and slightly displaced distally with respect to the lingual cusps. Three transverse cristids (metalophulid I, hypolophid and posterolophid) and one longitudinal cristid (ectolophid) are present, the latter of which meets an incipient posterior arm of the protoconid. Along the labial portion of the tooth the base is inflated, particularly around the protoconid, forming an incipient cingulum. The metalophulid I delimits the mesial wall of the crown and connects the mesiolingual side of the protoconid with the labial side of the metaconid. The anterior basin is wide and closed by a low lingual wall formed by the posterior arm of the metaconid that ends at the mesial aspect of the entoconid. The ectolophid is complete and courses from the protoconid to reach the junction of the hypolophid and the anterior arm of the hypoconid. The posterolophid runs from the hypoconid to meet the hypoconulid. The posterolophid has a robust labial portion, whereas its lingual portion tapers towards the entoconid. The hypoflexid is large and deep.

The second lower molar (M_2_) is similar in morphology to the first lower molar but differs in having more robust and well developed lophids (Figs. 7F–7G). Furthermore, the lingual wall, formed by the posterior arm of the metaconid and the anterior arm of the entoconid, is relatively taller than in M_1_. The labial cusps (protoconid and hypoconid) are larger and more labial in position than those on M_1_, with relatively less basal inflation of the enamel. The hypoconulid is weakly developed and there is no depression between the hypoconid and the hypoconulid.

There is only one maxillary specimen in the hypodigm (DPC 4275), a fragment of a left maxilla with dP^4^ and M^1^ and an alveolus for dP^3^ (Figs. 7A–7C). The infraorbital foramen is only partially preserved, but it is clearly broad and hystricomorphous. The margins of the incisive foramen cannot be traced with confidence due to damage in this area. The ventral ramus of the zygomatic process is thick. On the ventral view of the maxilla at the base of the ventral ramus of zygomatic process there is a ridge defining a broad fossa for the attachment of the superficial masseter.

The dP^4^ is somewhat trapezoidal in shape and broader labially than lingually (Fig. 7C). The crown of the tooth bears four major cusps (paracone, metacone, protocone and hypocone) and a well-developed metaconule. The protoloph is a well-developed, transverse cristid which courses labially from the submerged paracone and thins toward the labial portion of the protocone. The anteroloph is lower than the protoloph and runs from the labial aspect of the protocone to terminate near the mesial aspect of the paracone, delimiting a large paraflexus. There is no mesostyle. A small centrally-placed metaconule is connected to the hypocone *via* a short but robust anterior arm of the latter cusp. A very thin and incomplete mure is faintly visible. There is a remnant of a metaloph that turns mesially from the metacone to meet the metaconule, delimiting a deep but small fovea (posterofossette) on the distal portion of the tooth. From the hypocone, the posteroloph runs labially to connect to the base of the metacone. The posteroloph is relatively weakly developed when compared with the anteroloph. The labial wall is formed by a long posterior arm of the paracone that terminates at the base of the metacone. The hypoflexus is deep and no endoloph is present. There is a very small accessory cusp (enterostyle?) in the distolingual portion of the sinus. The M^1^ has the same basic occlusal configuration as dP^4^, but is larger, is transversely broader, has no mure, and has relatively well-developed cusps, including an incipient anterostyle (Fig. 7C).

***Comparison of* T. lavocati *with other possible phiocricetomyines***

The phylogenetic analyses of Marivaux & Boivin (2019) placed ten taxa other than *T. lavocati* within Phiocricetomyinae, significantly expanding the possible membership of the subfamily (Table 2). Here we expand our comparisons with these possible relatives of *T. lavocati*.

**Table 2:** Possible members of Phiocricetomyinae based on the phylogenetic results of Marivaux & Boivin (2019).

Age	taxon	Locality	Reference
late middle Eocene	“*Protophiomys” tunisiensis*	Tunisia	Marivaux et al. (2014)
late Eocene	*Talahphiomys libycus*	Libya	Jaeger et al. (2010)
latest Eocene	*Birkamys korai*	Egypt	Sallam & Seiffert (2016)
latest Eocene	*Mubhammys vadumensis*	Egypt	Sallam & Seiffert (2016)
earliest Oligocene	*Mubhammys atlanticus*	Morocco	Marivaux et al. (2017)
earliest Oligocene	*Neophiomys minutus*	Morocco	Marivaux et al. (2017)
earliest Oligocene	*Phenacophiomys occidentalis*	Morocco	Marivaux et al. (2017)
early Oligocene	*Neophiomys paraphiomyoides*	Egypt and Libya	Coster et al. (2012) and Wood (1968)
early Oligocene	*Neophiomys dawsonae*	Libya	Coster et al. (2012)
early Oligocene	*Phiocricetomys minutus*	Egypt	Wood (1968)

*T. lavocati* differs from *Birkamys* in having a more posteriorly placed mental foramen; smaller metaconids and entoconids relative to protoconids and hypoconids; relatively well developed dP_4_-M_2_ hypoconulids; a more distally placed dP_4_ protoconid; and in lacking M_1–2_ anterior cingulids. The dP^4^-M^1^ of *T. lavocati* differ from those of *Birkamys* in having a distinct metaconule (rather than being submerged into the mure); more labially placed protocones and hypocones, particularly on dP^4^; relatively trenchant posterolophs; and in lacking a distinct metaloph on M^1^. *T. lavocati* differs from *Neophiomys minutus* in having little or no development of the metalophulid II on M_1–2_; well-developed dP^4^ metaconule; a relatively large dP^4^ paraflexus; a relatively well developed posterior arm of the paracone on dP^4^; and a relatively weak and more labially placed mure on dP^4^. *T. lavocati* differs from *Neophiomys dawsonae* in being relatively small and in having little or no development of the metalophulid II on M_1–2_; a well-developed metaconule on M^1^; and no M^1^ mure or metaloph. *T. lavocati* differs from *Neophiomys paraphiomyoides* in being smaller; in having an anteroconid on dP_4_, and in lacking any development of metalophulid II on the lower molars. The M^1^ of *T. lavocati* differs in having a well-developed metaconule, and in lacking a mure and a distinct metaloph. *T. lavocati* is smaller than *Mubhammys*, and differs in having a more posteriorly placed mental foramen; a more distally placed dP_4_ protoconid and relatively short dP_4_ (but more trenchant) ectolophid; lingually closed M_1–2_ mesoflexids; relatively well developed dP^4^-M^1^ metaconules; no mesostyles on dP^4^-M^1^; and a metaloph, anterostyle, and more distinct anteroloph on dP^4^. *T. lavocati* is smaller than *Phenacophiomys occidentalis* and differs in having labial cusps larger in size than the lingual cusps; a more distally placed dP_4_ protoconid and relatively short dP_4_ ectolophid; and no anterior cingulid on dP_4_-M_1_. *T. lavocati* further differs in lacking any connection between the metaloph and the metaconule on M^1^ and in having no development of metalophulid II on dP_4_-M_2_. *T. lavocati* differs substantially from “*Protophiomys*” *tunisiensis* in having more bunodont cusps and more robust crests; lingually closed mesoflexids; no development of metalophulid II on dP_4_-M_2_; no anterior cingulids on dP_4_-M_2_; no connection between the metaloph and metaconule on M^1^; and no development of a mesostyle on M^1^.

Finally, compared to early Oligocene *Phiomys andrewsi* (the type species of *Phiomys*), *T. lavocati* is smaller in size, and has a mental foramen at the level of the premolar rather than mesial to it; a relatively short ectolophid; more bulbous cusps; and lacks a mesoconid, metalophulid II, posterior arm of protoconid, and anterior cingulid. In the upper dentition, *T. lavocati* differs from *P. andrewsi* in having a well-developed metaconule and anteroloph, and in lacking a mesolophule; on dP^4^ the protocone and the hypocone are more labially placed.

**Remarks**

Based on morphological and metric grounds (Figs. 3–6), we are able to demonstrate that *T. lavocati* is restricted to Quarry E in the lower sequence of the Jebel Qatrani Formation of northern Egypt. The specimens referred to *T. lavocati* from the Libyan sites DT-LOC-2 and ZR5 by Jaeger et al. (2010) and Coster et al. (2012), respectively, do not belong to that species and are now in need of revision. Among other things, with the new information provided by *Qatranimys safroutus* (see below), we do not consider the specimen identified by Jaeger et al. (2010; p. 206; Fig. 6M) as an M^2^ of *T. lavocati* (DT-2-103) to be an M^2^ (we identify it as an M^1^) or to belong to *T. lavocati*.

**Family** *Incertae sedis*

**Subfamily** Phiocricetomyinae Lavocat, 1973

**Figure 8:**
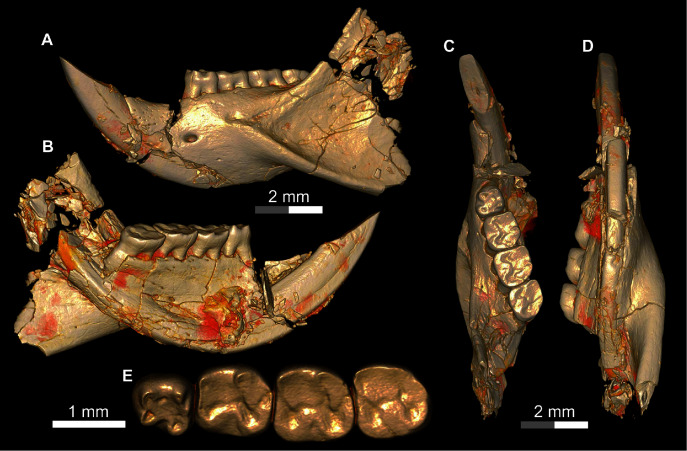
DPC 17813, complete left mandible with I and P_4_-M_3_ of *Qatranimys safroutus*, new genus and species, from Quarry L-41. (A) Lateral, (B) medial, (C) occlusal, and (D) ventral views; and (E) occlusal surface. Each of the grey and/or white division in the scale bar refers to 1 mm.

**Figure 9:**
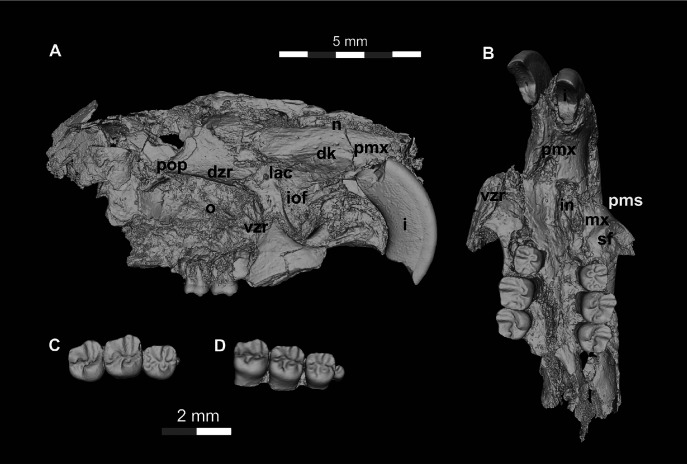
DPC 16815, cranium of *Qatranimys safroutus* (new genus and species) from Quarry L-41. (A) Lateral view of right side; (B) inferior view; (C) dP^4^-M^2^ of left side (reversed), and (D) dP^3^-M^2^ of right side. dk = dorsal bony keel, dzr = dorsal zygomatic ramus, i = incisor, iof = infraorbital foramen, o = orbit, pms = premaxilla–maxilla suture, pmx = premaxilla, mx = maxilla, pop = postorbital process, vzr = ventral zygomatic ramus, n = nasal, lac = lacrimal, in = incisive foramen. Each of the grey and/or white divisions in the scale bar represents 1 mm.

**Figure 10:**
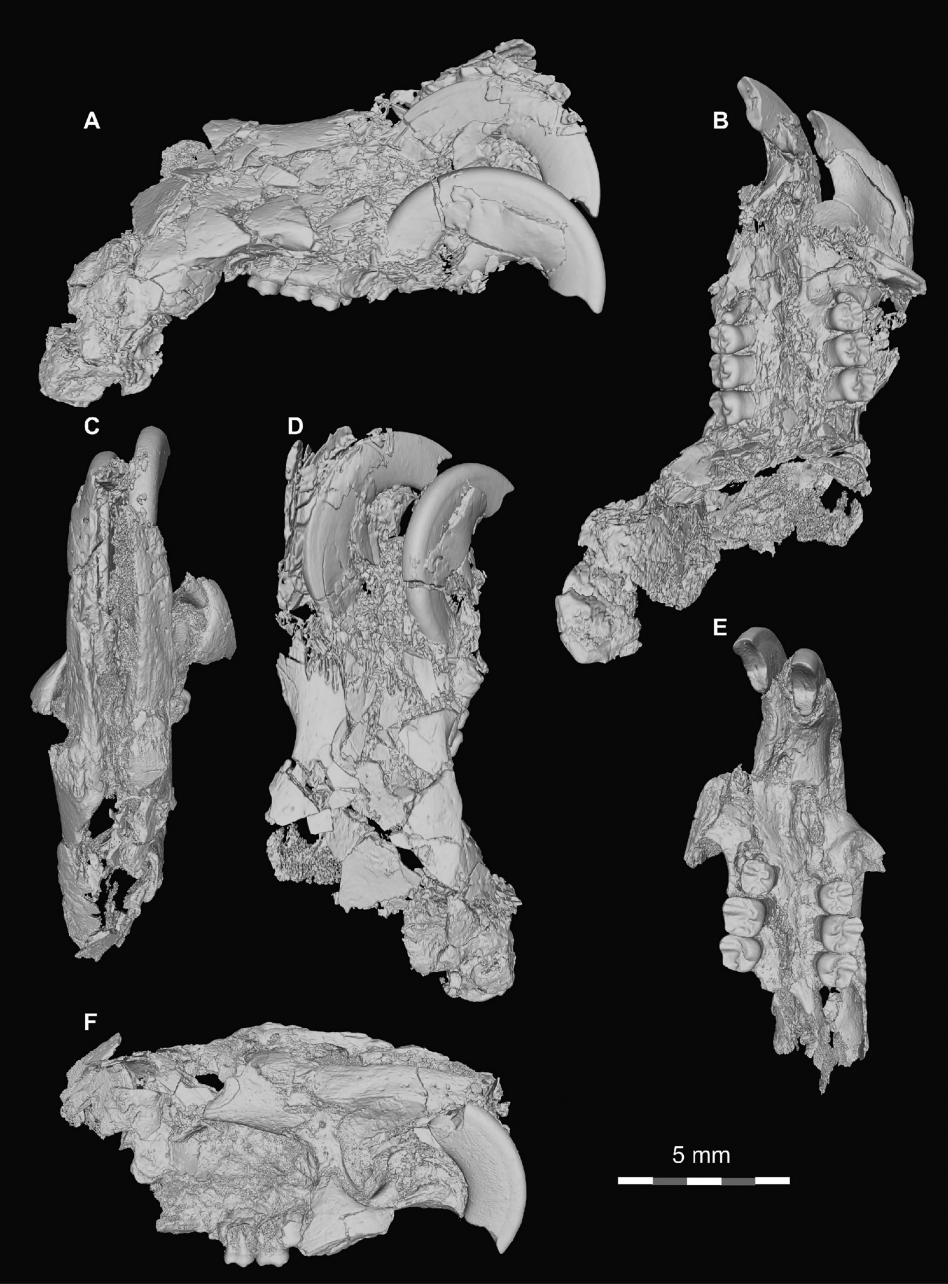
DPC 10300 and DPC 16815, crania of *Qatranimys safroutus* (new genus and species) from Quarry L-41. (A) Lateral view of right side of DPC 10300; (B) inferior view of DPC 10300; (C) dorsal view of DPC 16815; (D) dorsal view of DPC 10300; (E) inferior view of DPC 10300; (F) lateral view of right side of DPC 16815. Each of the grey and/or white divisions in the scale bar represents 1 mm.

*Qatranimys*, new genus urn:lsid:zoobank.org:act:3866127A-97CF-43A1-B7DC-F042805AE197 (Figs. 8–10)

***Type and only species***

*Qatranimys safroutus*, new species urn:lsid:zoobank.org:act:65B76CA2-42C9-4674-8C2A-A146994DAD3B

***Etymology***

Combination of ‘qatrani’, Arabic for tar, and in reference to the Jebel Qatrani (“tar hills”) region where the species is found, and ‘mys’, Greek meaning mouse.

***Diagnosis***

As for the type and only species.

*Qatranimys safroutus*, new species urn:lsid:zoobank.org:act:[ID] (Figs. 3 and 5)

***Etymology***

From colloquial Egyptian Arabic *safrout* (سفروت), meaning tiny.

***Diagnosis***

*Q. safroutus* differs from *T. lavocati* in having a relatively short dP_4_ with a metalophulid I; no inflation of the enamel surrounding the bases of the protoconid and hypoconid on M_1–2_ (and no incipient cingulid around the M_1–2_ protoconids); an M_1_ that is, on average, smaller than M_2_ (see bivariate plot in Fig. 6); a lingually closed M_2_ metaflexid, with a relatively high connection of the posterolophid to the entoconid; mesial and lingual margins of the mesiolingual corner of M_1–2_ that form a relatively obtuse angle, rather than a ~90 degree angle; a relatively short dP^4^ with a labial margin approximately equal in length to the lingual margin, and with a smaller paraflexus, a more distally placed hypocone, and a relatively buccally placed paracone; and an M^1^ with a relatively narrow distal moiety. The M_1_ of *Q. safroutus* differs from the possible M_1_ of *T. libycus* (identified as an M_2_ by Jaeger et al., 2010; p. 203; Fig. 5X) in lacking an anterior cingulid, and in having a more obtuse angle between the mesial and lingual margins of the crown and a shorter posterior arm of the metaconid. The M^1^ of *Q. safroutus* differs from that of *T. libycus* in lacking both a mesostyle and a long mesolophule that meets the buccal margin of the tooth, and in having a larger anterostyle, a metaloph that is curved toward the metaconule, and a lingually positioned metacone. The M^2^ of *Q. safroutus* differs from the M^2^ of *T. libycus* in having a low mure, a relatively well developed metaconule, and a relatively small metacone. *Q. safroutus* differs from the “*T. lavocati*” specimens from Dur at-Talah Locality (DT-LOC-2) (Figs. 4–5) in having a more distally placed dP_4_ protoconid, with a distinct metalophulid I; M_1–2_ with different proportions (being mesiodistally shorter than buccolingually broad; see bivariate plot in Fig. 6); a relatively well developed posterior arm of the metaconid and anterior arm of the entoconid on M_1–2_, closing off the mesoflexid lingually; no anterior cingulid on M_1–2_; a more obtuse angle between the mesial and lingual margins of M_1_; dP^4^ that is relatively broad in occlusal outline, with a more restricted paraflexus; a connection of the dP^4^ metaloph with the metaconule; a mure connecting to the protoloph on dP^4^; M^1^ that is relatively broad, with a connection between the metacone and metaconule, and an incipient mure.

***Holotype***

CGM 83743, right mandible with P_4_-M_3_ (Fig. 3F).

***Hypodigm***

The holotype; DPC 8825, right mandible with P_4_ and M_1–2_; DPC 10300, rostrum with right and left upper incisors and dP^3^-M^2^; DPC 14243, partial right edentulous maxilla; DPC 16815, cranium with two incisors, right dP^4^-M^2^ and left dP^3^-M^2^; DPC 20965, right maxilla with dP^3–4^; DPC 10710, left mandible with P_4_-M_3_; DPC 11345, left mandible with M_3_; DPC 14056, left mandible with M_1-3_; DPC 14187, right mandible with dP_4_-M_2_ and incisor; DPC 14393, left mandible with P_4_-M_2_; CGM 83743, right mandible with P_4_-M_3_; DPC 17813, right mandible with P_4_-M_3_ and incisor; DPC 17947, left mandible with dP_4_-M_2_ and incisor; DPC 20659, right mandible with M_1–3_; DPC 21818, left M_2–3_ (See Table 3 & Data S1).

**Table 3:** Mesiodistal length and buccolingual width of teeth (in millimeters) in the hypodigm of *Qatranimys safroutus* from Quarry L-41 of the Jebel Qatrani Formation.

Specimen	Side	Upper teeth									
		dP^3^		dP^4^		M^1^		M^2^		M^3^	
		Length	Width	Length	Width	Length	Width	Length	Width	Length	Width
DPC 10300	right	0.40	0.43	0.98	1.03	1.14	1.23	1.14	1.32	–	–
	left	0.37	0.40	0.98	1.00	1.12	1.23	1.13	1.34	–	–
DPC 16815	right	–	–	0.96	0.97	1.11	1.20	1.05	1.22	–	–
	left	0.40	0.43	0.98	0.98	1.10	1.20	1.04	1.22	–	–
DPC 20965	right	0.56	0.60	1.34	1.29	–	–	–	–	–	–
No.		4		5		4		4		0	
Mean		0.433	0.465	1.048	1.054	1.117	1.215	1.09	1.275	–	–
		Lower teeth									
		dP_4_		P_4_		M_1_		M_2_		M_3_	
		Length	Width	Length	Width	Length	Width	Length	Width	Length	Width
CGM 83743	right	–	–	1.03	0.90	1.17	1.08	1.19	1.08	1.24	1.05
DPC 8825	right	–	–	0.95	0.88	1.14	1.07	1.19	1.05	–	–
DPC 10710	left	–	–	0.96	0.91	1.16	1.09	1.13	1.08	1.22	1.14
DPC 11345	left	–	–	–	–	–	–	1.24	1.16	1.28	1.17
DPC 14056	left	–	–	–	–	1.14	1.1	1.24	1.21	1.08	1.00
DPC 14187	right	1.01	0.78	–	–	1.11	0.99	1.22	1.17	–	–
DPC 14393	left	–	–	0.98	0.96	1.13	1.11	1.15	1.13	–	–
DPC 17813	right	–	–	0.91	0.91	1.09	1.04	1.15	1.12	1.09	1.09
DPC 17947	left	0.99	0.76	–	–	1.04	1.0	1.18	1.12	–	–
DPC 20659	right	–	–	–	–	1.08	1.16	1.20	1.17	0.99	1.0
DPC 21818	left	–	–	–	–	–	–	1.21	1.11	1.1	0.98
No.		2		5		9		11		7	
Mean		1.0	0.770	0.966	0.912	1.118	1.071	1.191	1.127	1.143	1.061

***Type locality***

Locality 41, Jebel Qatrani Formation, Fayum Depression, Egypt.

***Description***

The mandible is similar to those of other Fayum hystricognaths in having an angular process that is placed lateral to the plane of the incisor and tooth row, leaving a wide groove between the angular process and the incisor alveolus in ventral view; this area provides the insertion for the *pars reflexa* of the superficial masseter muscle (Hautier & Saksiri, 2009). The ascending ramus is posteriorly inclined and originates lateral to the alveolar plane near the base of the M_1_ and M_2_ (as in DPC 17813, Fig. 8). The tip of the coronoid process is not preserved. The horizontal ramus is robust and ventrally convex, with its deepest point being below the P_4_. The diastema is slightly deeper than the alveolar plane. On the lateral surface, the mental foramen is relatively small and varies from being oval to round in outline; it is situated directly under the mesial part of P_4_. The masseteric fossa is deep, posteriorly broad and tapering anteriorly to terminate below the M_1_. The dorsal masseteric ridge is weakly developed and crosses the dorsal surface of the horizontal ramus under the posterior part of M_1_. There are some nutrient foramina scattered on the horizontal ramus. The ventral masseteric ridge is well developed, originates laterally from the area beneath the anterior part of M_1_, and continues posteroventrally towards the angular process, which is not preserved (Fig. 8A). On the medial side, the angular process initiates beneath the area of M_3_. The mandibular foramen is not preserved. The symphysis is partially preserved in DPC 14056 and DPC 17947.

The lower incisor is well preserved in many specimens. The tip of the incisor projects above the tooth row and extends distally to terminate posterior to M_3_ (Fig. 8B). It is covered anteriorly by smooth enamel that extends to the labial and lingual sides, covering about one third and one fourth of the labial and lingual sides of the incisor, respectively, as seen in all Fayum hystricognaths. On the occlusal surface, the pulp cavity is preserved, has an oval shape, and is posteriorly placed.

The dP_4_ is only known from two specimens in the hypodigm (DPC 17947 and DPC 14187) (Figs. 3C and 3D). The P_4_ is present in several specimens, indicating that dP_4_ is replaced by the permanent premolar. The dP_4_ is generally pear-shaped in outline and longer mesiodistally than labiolingually. The occlusal pattern is trilophodont (with metalophulid I, hypolophid and posterolophid) and displays five major cusps (metaconid, protoconid, entoconid, hypoconid and hypoconulid) that are more or less equal in size and are of the same height. On the mesial portion of the tooth the protoconid is distal in position with respect to the metaconid. The protoconid and metaconid are connected *via* the metalophulid I which is arc-shaped and runs mesiolingually, delimiting the posterior wall of a broad mesial shelf. On this shelf, there is a prominent isolated anteroconid mesiolabial to the metaconid. The lingual wall between the metaconid and the entoconid is low, leaving the wide and deep mesoflexid closed lingually. In DPC 17947 (Fig. 3C), there is an incipient mesostylid near the mesial aspect of the entoconid. The ectolophid is short, low relative to the cusp height, and joins the protoconid to the junction of the anterior arm of the hypoconid and the hypolophid. The anterior arm of the hypoconid is short and connects to a well-developed hypolophid. The latter extends lingually to connect with the entoconid. On the distal portion of the crown, the entoconid is placed mesially with respect to the hypoconid. The hypoconulid is a well-developed cusp on the middle of the posterolophid forming the very distal portion of the tooth. The posterolophid runs from the hypoconid to end and taper distolabial to the base of entoconid, leaving the posterior basin opened lingually.

Five specimens preserve the P_4_ (CGM 83743, DPC 8825, DPC 10710, DPC 14393, and DPC 17813). The tooth is relatively shorter and broader when compared with the dP_4_. It is roughly rectangular to square in shape, with the talonid slightly wider than the trigonid and bearing four main cusps (metaconid, entoconid, protoconid, and hypoconid). The metaconid and entoconid are placed roughly transverse to the protoconid and hypoconid, respectively. The metalophulid I is complete in DPC 8825 and DPC 17813, but in CGM 83743, DPC 10710, and DPC 14393 the metalophulid I is interrupted by a narrow notch lingual to the protoconid. In DPC 14393 (Fig. 3G) the metaconid and the protoconid are more bulbous and there is an incipient anteroconid mesial to the protoconid. There is no hint of the posterior arm of the protoconid in any of the specimens. In CGM 83743 (Fig. 3F) and DPC 14393 (Fig. 3G), the anterior basin of the P_4_ is generally large and open lingually *via* a deep notch on the lingual wall. The other specimens have a low lingual wall closing the mesoflexid. The hypoconid has a well developed anterior arm that is connected to a well developed ectolophid. The hypolophid shows considerable variability—in DPC 8825, DPC 14393 and DPC 17813, the hypolophid arcs distolabially to form a direct connection with the posterolophid, delimiting a small fovea, while in DPC 10710 (Fig. 3E) the hypolophid is complete, connecting to the anterior arm of the hypoconid. In CGM 83743 (Fig. 3F), the hypolophid extends from the entoconid toward the anterior arm of the hypoconid but it ends abruptly at the center of the tooth, connecting the posterior basin with the central basin *via* a notch. On the distal portion of the crown, the hypoconulid varies from being small to distinct and is subsumed into a posterolophid that terminates at the distal aspect of the entoconid, delimiting the posterior margin of the tooth.

The M_1_ is roughly rectangular in shape, with almost all specimens being slightly longer than wide. The tooth has five bulbous cusps (protoconid, metaconid, entoconid, hypoconid and hypoconulid) and three transverse cristids in the occlusal pattern (metalophulid I, hypolophid, and posterolophid). The metaconid is placed transverse to the protoconid, while the entoconid is situated mesial to the hypoconid. The metalophulid II is not present, with only a small knob protruding from the lingual face of the protoconid. The metalophulid I runs labially from the metaconid to reach the mesiolingual side of the protoconid. In DPC 14056 (Fig. 3I), the metalophulid I is interrupted by a small notch. The hypolophid is well developed and attaches to the anterior arm of the hypoconid near that crest’s junction with the ectolophid. The ectolophid is well developed and situated near the middle, or just labial to the middle, of the tooth. The posterolophid runs distolingually from the hypoconid and terminates at the base of the distal aspect of the entoconid, delimiting the posterior margin of the tooth. The hypoconulid is well developed and more or less the same size as the hypoconid. The two lingual cusps (metaconid and entoconid) are relatively small compared to the labial cusps. The mesoflexid is broader and delimited by a lingual wall formed by the posterior arm of the metaconid that reaches the mesial aspect of the entoconid. The hypoflexid is transversely wide and deep.

The M_2_ is quadrangular with a rounded posterior portion. The occlusal surface of the M_2_ is similar to that of the M_1_ but differs in being slightly larger in size, and having more developed transverse lophids, a broader trigonid relative to the talonid, a taller posterolophid and lingual wall of the trigonid, a less distinct hypoconulid, and a more lingually placed metaconid.

The M_3_ is preserved in six specimens (DPC 10710, DPC 11345, DPC 14056, CGM 83743, DPC 17813 and DPC 20381). Some specimens are triangular in shape, with a talonid that is much narrower than the trigonid. Most M_3_s are smaller in size than M_2_s, however in two individuals (DPC 10710 and DPC 11345) the M_3_ is similar in size to the M_2_. Otherwise, the M_3_ has a very similar occlusal pattern to that of M_1_ and M_2_. The metalophulid II varies from being very short to absent. On DPC 11345, the metalophulid II of M_3_ runs lingually to reach the middle of the mesoflexid. The hypoconulid is submerged into the short posterolophid, delimiting the distal lobe of the crown.

The upper incisors are shorter and more highly arched than those of the mandible. They extend posteriorly to terminate just anterior to the dP^3^. The incisors are covered by smooth enamel which extends labially and medially to cover only one third and one fourth of both sides respectively. In the occlusal surface, in the middle of the dentine layer there is a pulp cavity with a slit shape.

The dP^3^ is well preserved in three specimens (DPC 16815, DPC 10300 and DPC 20965). It is a very small simple round tooth in occlusal view, with one main cusp that abuts the mesial surface of dP^4^. On DPC 10300 there is a small accessory cusp.

The dP^4^ varies from being square to slightly more trapezoidal in outline. The crown bears four major cusps (paracone, metacone, protocone and hypocone), all of which are more or less the same size and height. The anterostyle is small and situated mesial to the protocone, and is connected to that cusp by a short crest that runs from the mesiolingual part of the protocone. The anteroloph extends mesiolabially from the anterostyle and terminates near the mesial base of the paracone. The protoloph is a well-developed transverse crest. There is no hint of a parastyle, mesostyle, or mesolophule. The metaloph is short and turns mesiolingually from the metacone to meet the metaconule. The metaconule is situated near the center of the crown and is connected to the hypocone by a well-developed anterior arm of the hypocone. A mure is present and meets the protoloph labial to the protocone. The posteroloph is relatively low and runs labially from the hypocone to delimit the posterior margin of the tooth and connects with the distal aspect of the metacone. The labial wall is complete and relatively low as it does not reach the height of the two labial cusps (paracone and metacone).

The M^1^ is very similar in occlusal configuration to that of dP^4^, but is larger, relatively broad labiolingually, and has relatively well-developed lophs and cusps, including a relatively large anterostyle and a metacone that is relatively small and lingually positioned when compared to the paracone. The metaconule is weakly developed and the connection between the metacone and metaconule is either very faint or absent. The paraflexus is relatively small when compared with that of dP^4^. The M^2^ is broader than M1 and has a similar occlusal configuration, but the distolabial corner of the tooth is much different in having a reduced and somewhat crestiform metacone that lacks any hint of a metaloph; together with the anterior arm of the metacone and the posteroloph, the metacone encloses a large fossa comprised of a broadly open mesoflexus + posteroflexus.

The only other hystricognath crania from the Paleogene of Africa are also from Quarry L-41, so the description of the new skull elements of *Qatranimys* (Figs. 9 and 10) is based on comparison with the sympatric and synchronous *Gaudeamus* (Sallam, Seiffert & Simons, 2011), *Acritophiomys* (Sallam, Seiffert & Simons, 2012) and *Birkamys* (Sallam & Seiffert, 2016). As with most fossils from L-41, the new specimens are compressed and bear numerous surface cracks and displacements due to severe postmortem distortion. We figure as much as is possible through volume rendering of the skulls, using high-resolution micro-CT scans with minimal physical preparation of these small and fragile specimens. Four crushed cranial specimens have been recovered (DPC 10300, DPC 14324, DPC 16815 and DPC 20956). DPC 10300 is dorsoventrally crushed and includes most of the front of the cranium, including the premaxillae with two upper incisors, the frontals, both maxillae and the entire dentition aside from both M^3^s; DPC 16815 is mediolaterally crushed and preserves the snout (premaxillae and nasals), the maxillae, the frontals and the parietal in addition to complete dentition aside from both M^3^s; DPC 14243 is a maxilla with roots of dP^4^ and alveoli of dP^3^; and DPC 20956 is a maxilla that preserves the third and fourth premolars.

The nasal bones can only be seen clearly in DPC 16815, whereas in DPC 10300 they are highly deformed and extensively damaged. In DPC 16815, the paired nasals’ articular surfaces with the frontals extend to the level of the dorsal zygomatic ramus, above the level of the dP^4^ (as in *Gaudeamus*). In DPC 10300, the bones extend backward to articulate with the frontals at the level of M^1^-M^2^ posterior to the infraorbital foramen (due to post-mortem displacement). The articular relationships between the nasals and the premaxillae are obscured by distortion.

The premaxillae house two upper incisors, form the upper diastema, and contribute to much of the rostrum. The posterodorsal processes of the premaxilla are preserved in DPC 16815 but missing in DPC 10300. On the lateral side of the posterodorsal process, there is a bony keel protruding dorsally. In lateral view the premaxillae decrease in width anteriorly leading to an arched diastema as in *Gaudeamus* and *Acritophiomys*. The premaxilla is bounded posterolaterally by the maxilla. Despite the postmortem distortion, the borders of the incisive foramen can be clearly seen in DPC 10300 (Fig. 10B), showing that it was large and elongate and likely formed an “anterior palatine fenestra” as in *Acritophiomys*, *Birkamys*, *Gaudeamus*, *Mubhammys*, and *Waslamys* (Sallam & Seiffert, 2016). The postorbital processes are present but very small, unlike *Gaudeamus* which has prominent processes oriented laterally and posteriorly; in *Qatranimys*, the process has a distinct vascular foramen on its underside, observable on both sides of DPC 10300. The approximate outline of both orbits is preserved on both sides of DPC 16815, but details of the orbital mosaic are impossible to determine due to breakage. The jugal is not preserved in any of the specimens. The suture between the lacrimal bone and the posterodorsal process of the premaxilla, and that with the dorsal zygomatic ramus is preserved on both sides of DPC 16815, however the outline of the lacrimal bone cannot be determined with confidence. The dorsal exposure of the lacrimal bears a small foramen on the right and left sides of DPC 16815 (Fig. 9A). The lacrimal foramen is relatively large and is situated in the middle of the bone.

In lateral view, the maxilla contributes to the anterior wall of the orbit, moreover the facial process of the maxilla joins with the posterior portion of the premaxilla to form the lateral wall of the rostrum, and the medial portion of the infraorbital foramen. All of the crania have an enlarged (hystricomorphous) infraorbital foramen, through which the medial masseter muscle *pars anterior* extends (Hautier & Saksiri, 2009). The infraorbital foramen shows a ventrolaterally rounded outline, as seen on the left side of DPC 16815 (Fig. 9A). The ventral ramus of the zygomatic process of the maxilla extends laterally from the area in front of the dP^3^ and then arches posteriorly, delimiting the anteroventral portion of the orbital margin. The anteroventral portion of the ventral zygomatic ramus bears a deep fossa for the insertion of the superficial masseter muscle, and, posteriorly, a relatively shallow fossa for the origin of the lateral masseter, as in *Gaudeamus* and *Acritophiomys*. The dorsal zygomatic ramus is oriented dorsoventrally in DPC 16815 (Fig. 9A). As in *Gaudeamus*, the roots of the ventral and dorsal rami extend anteriorly to roughly the same point. It is difficult to trace the original morphology of the palate due to damage, however it appears to be flat and broad. The parietal bones are poorly preserved in DPC 16815, whereas in DPC 10300 they are completely missing (Fig. 10).

***Comparison of* Q. safroutus *with other possible phiocricetomyines***

*Q. safroutus* differs from *Birkamys* in having a more posteriorly placed mental foramen; smaller metaconids and entoconids relative to protoconids and hypoconids; relatively well developed dP_4_-M_2_ hypoconulids; a more distally placed dP_4_ protoconid; and in lacking M_1–2_ anterior cingulids. The dP^4^-M^2^ of *Q. safroutus* differ from those of *Birkamys* in having distinct metaconules (rather than being submerged into the mure) and more robust primary cusps. *Q. safroutus* differs from *Neophiomys minutus* in having relatively weak development of the metalophulid II on M_1–2_; well-developed dP^4^ metaconules; a relatively large dP^4^ paraflexus; a relatively well developed posterior arm of the paracone on dP^4^; and a relatively weak and more labially placed mure on dP^4^. *Q. safroutus* differs from *Neophiomys paraphiomyoides* in having a distinct anteroconid, a broad mesial shelf, and smaller cusps on dP_4_. The metaconid of dP_4_ is anteriorly placed relative to the protoconid, rather than being buccolingually opposed. The two latter cusps are connected by a relatively long metalophulid I rather than being closely positioned. The M_1–2_ of *Q. safroutus* differ from those of *N. paraphiomyoides* in having no development of metalophulid II or anterocingulid in M_1–2_ and in having a well-developed hypoconulid on M_2_. The upper molars differ by exhibiting a distinct central metaconule, a relatively weak mure and no development of a metaloph. *Q. safroutus* differs from *Neophiomys dawsonae* in having a distinct metaconule, labial and lingual walls on upper and lower molars respectively, no development of the metalophulid II and no anterior cingulid. *Q. safroutus* is smaller than *Mubhammys vadumensis*, and differs in having a more posteriorly placed mental foramen; lingually closed mesoflexids; and mesial and lingual margins of the mesiolingual corner of M_1–2_ that form an obtuse angle and no development of anterior cingulids. In dP_4_, *Q. safroutus* differs in having a more distally placed protoconid; a broad mesial shelf; a well-developed anteroconid; and a relatively short but robust ectolophid and relatively long metalophulid I. In the upper molars, *Q. safroutus* differs in having relatively well developed dP^4^-M^1^ metaconules; labially closed paraflexus (parafossette); no mesostyles; and a weak mure. *Q. safroutus* differs further by having a metaloph, anterostyle, and more distinct anteroloph on dP^4^. *Q. safroutus* differs substantially from *Phenacophiomys occidentalis* by being smaller, and in having labial cusps larger in size than the lingual cusps; a more distally placed protoconid and relatively short ectolophid in dP_4_; and no anterior cingulid or any development of metalophulid II on lower molars. In the upper molars, *Q. safroutus* differs in lacking mesostyle. *Q. safroutus* differs from “*Protophiomys”* *tunisiensis* in having a well-developed metalophulid I connecting the protoconid with metaconid on the dP^4^ rather than being separated. The lower molars of *Q. safroutus* differ further in having a lingual wall; no development of the metalophulid II; and no anterior cingulids. Furthermore, M^1^ shows no development of a mesostyle.

## Discussion

We detected significant morphological differences between *T. lavocati* from Fayum Quarry E and the “*T. lavocati*” specimens from the Libyan sites DT-LOC-2 and ZR5 by Jaeger et al. (2010) and Coster et al. (2012), respectively. Among other features, the bases of the protoconid and the hypoconid in M_1–2_ of *T. lavocati* are basally inflated, forming an incipient cingulid. Furthermore, the mesoflexids are closed lingually *via* the posterior arm of the metaconid and the anterior arm of the entoconid, and there are no mesostyles or mesolophules in the upper molars. The bivariate plot of M_1_ and M_2_ proportions (Fig. 6) shows broad overlap in M_1_ relative to M_2_ in *T. lavocati* from Quarry E, and obvious size differences from both the late Eocene (Dur at-Talah) and early Oligocene (Zallah) “*T. lavocati*”.

Jaeger et al. (2010) suggested that the rodent layers from Dur at-Talah correlate with Chron 18n (~39 and 38 Ma; Upper Bartonian)—several million years older than the *T. lavocati* type locality Quarry E, which is placed near the bottom of Chron C12r (Seiffert, 2006), at ~31–33.2 Ma (*i.e*. Rupelian). If the date that Jaeger et al. (2010) proposed for the Dur at-Talah sites, and identification of *T. lavocati* at those sites, were correct, it would require the species duration for *T. lavocati* to be, at a minimum, 7 million years long. Oddly, Jaeger et al. (2010) never acknowledge this discrepancy and its biochronological implications, and instead focused their discussion entirely on evidence that they considered to support an older rather than a younger age for the sites. Sallam & Seiffert (2016) helped to resolve this paradox by using a Bayesian tip-dating approach to estimate the ages of the Dur at-Talah rodents, and found that the sites more likely date to the late Eocene—*i.e*. intermediate in age between the Fayum BQ-2 and L-41 localities, and several million years younger than the ages proposed by Jaeger et al. (2010). This late Eocene age for the Dur at-Talah rodents has also been accepted by Marivaux et al. (2017).

Given the morphological and metric grounds provided in this work, in addition to stratigraphic range, it can now be demonstrated that the specimens from the Libyan sites actually do not belong in the species *lavocati*. Hence, the specimens from Dur at-Talah and Zallah placed in “*T. lavocati*” should be revised in the future. Moreover, the Fayum Quarry E in the lower sequence of the Jebel Qatrani Formation is considered to be the type and only locality of *T. lavocati*. Nevertheless, we maintain the genus name *Talahphiomys* as the generic replacement name for “*Phiomys*” *lavocati* as this replacement name has priority.

We note interesting points of similarity between *T. lavocati* and the enigmatic early Oligocene *Phiocricetomys* (Wood, 1968) that are not seen in the Libyan “*T. lavocati*” material, namely the development of an incipient labial cingulid around the M_1_ protoconid in *T. lavocati*—likely presaging the large and well-developed M_1_ labial cingulid seen in *P. minutus*. They also share a deep sulcus separating the isolated dP_4_ protoconid from the anteroconid and metaconid. Future phylogenetic analyses that take into account all existing Paleogene Afro-Arabian hystricognaths can test the hypothesis that *T. lavocati* is the exclusive phiocricetomyine sister taxon of *Phiocricetomys*.

The tiny species *Qatranimys safroutus* from Quarry L-41 is one of the most diminutive rodent fossils known. *Q. safroutus* is presumably more primitive than *T. lavocati* in having no inflation in the labial part of M_1–2_, and in retaining metalophulid I in dP_4_. For the first time, the large sample of *Q. safroutus* allows for an understanding of natural intraspecific variation within a phiocricetomyine species, and bolsters the case for the Libyan “*T. lavocati*” not being conspecific with either *T. lavocati* or *Q. safroutus*.

The phylogenetic position of phiocricetomyines relative to Phiomorpha remains a matter of uncertainty. Many studies placed them outside of the Phiomorpha-Caviomorpha clade (Sallam et al., 2009; Sallam, Seiffert & Simons, 2011; Sallam & Seiffert, 2016; Marivaux & Boivin, 2019) while others placed them as stem phiomorphs (Sallam & Seiffert, 2016, 2019). Future phylogenetic analyses that aim to test these alternate hypotheses can now take into account cranio-mandibular features in addition to dental characters.

## Conclusions

In summary, our analysis of the available material from the Fayum and Dur at-Talah suggests that the Fayum Quarry E is the type and only locality of *T. lavocati*, and that the specimens from Dur at-Talah and Zallah do not belong to this species. Several features suggest that *T. lavocati* may be the exclusive phiocricetomyine sister taxon of *Phiocricetomys*. The vast material of the late Eocene *Q. safroutus* further supports the exclusion of the Libyan “*T. lavocati*” from both *T. lavocati* and *Q. safroutus*, the latter of which shows some primitive features when compared with *T. lavocati*. The dental features of the diminutive *Q. safroutus* further expand our understanding of the interspecific variation that might be expected among phiocricetomyine species. However, in order to develop a more detailed scenario for the membership of Phiocricetomyinae and relationships among known species, a more extensive phylogenetic analysis with both cranio-mandibular and dental characters will be needed.

## Supplemental Information

10.7717/peerj.12074/supp-1 Supplemental Information 1 Specimens links’ IDs for digital models on MorphoSource.

## Institutional abbreviations

CGM: Egyptian Geological Museum, Cairo, Egypt
DPC: Duke Lemur Center Museum of Natural History, Duke University, Durham, North Carolina, USA
YPM: Yale Peabody Museum of Natural History, Yale University, New Haven, United States

## Additional Information and Declarations

### Data Availability

The following information was supplied regarding data availability: The specimens are deposited in the Egyptian Geological Museum, Cairo, Egypt (CGM) and the Duke Lemur Center Museum of Natural History, Duke University, Durham, North Carolina, USA (DPC). The specimen digital models are available at MorphoSource: CGM 83743, https://doi.org/10.17602/M2/M159418; DPC 4275, https://doi.org/10.17602/M2/M159418; DPC 5057, https://doi.org/10.17602/M2/M159377; DPC 8181, https://doi.org/10.17602/M2/M159381; DPC 8825, https://doi.org/10.17602/M2/M159383; DPC 10300, https://doi.org/10.17602/M2/M159387; DPC 10710, https://doi.org/10.17602/M2/M159393; DPC 11345, https://doi.org/10.17602/M2/M159402; DPC 14056, https://doi.org/10.17602/M2/M159404; DPC 14187, https://doi.org/10.17602/M2/M159408; DPC 14243, https://doi.org/10.17602/M2/M159409; DPC 14393, https://doi.org/10.17602/M2/M159416; DPC 16815 https://doi.org/10.17602/M2/M382303; DPC 17813, https://doi.org/10.17602/M2/M159421; DPC 17947, https://doi.org/10.17602/M2/M159422; DPC 20965, https://doi.org/10.17602/M2/M159436; DPC 21818, https://doi.org/10.17602/M2/M159425.

### New Species Registration

The following information was supplied regarding the registration of a newly described species: Publication LSID: urn:lsid:zoobank.org:pub:79E437BD-03EA-42BD-B341-D77EF2AC37F7 *Qatranimys* LSID: urn:lsid:zoobank.org:act:3866127A-97CF-43A1-B7DC-F042805AE197 *Qatranimys safroutus* LSID: urn:lsid:zoobank.org:act:65B76CA2-42C9-4674-8C2A-A146994DAD3B.
