# Supplementary material for: New phiocricetomyine rodents (Hystricognathi) from the Jebel Qatrani Formation, Fayum Depression, Egypt
Source: PeerJ. 2021 Oct 19;9:e12074. doi: 10.7717/peerj.12074 (PMC8533026; doi:10.7717/peerj.12074)
Supplement: Supplemental Information 1 [file peerj-09-12074-s001.docx]

**S1.** Specimens link IDs for digital models on MorphoSource.

| Specimens no. | ID for digital model on MorphoSource link |
| --- | --- |
| CGM 83743 | https://www.morphosource.org/Detail/MediaDetail/Show/media_id/82436 |
| DPC 4275 | https://www.morphosource.org/Detail/MediaDetail/Show/media_id/82489 |
| DPC 5057 | https://www.morphosource.org/Detail/MediaDetail/Show/media_id/82423 |
| DPC 8181 | https://www.morphosource.org/Detail/MediaDetail/Show/media_id/82424 |
| DPC 8825 | https://www.morphosource.org/Detail/MediaDetail/Show/media_id/82425 |
| DPC 10300 | https://www.morphosource.org/Detail/MediaDetail/Show/media_id/82426 |
| DPC 10710 | https://www.morphosource.org/Detail/MediaDetail/Show/media_id/82428 |
| DPC 11345 | https://www.morphosource.org/Detail/MediaDetail/Show/media_id/82431 |
| DPC 14056 | https://www.morphosource.org/Detail/MediaDetail/Show/media_id/82432 |
| DPC 14187 | https://www.morphosource.org/Detail/MediaDetail/Show/media_id/82433 |
| DPC 14243 | https://www.morphosource.org/Detail/MediaDetail/Show/media_id/82434 |
| DPC 14393 | https://www.morphosource.org/Detail/MediaDetail/Show/media_id/82435 |
| DPC 16815 | Scan in progress |
| DPC 17813 | https://www.morphosource.org/Detail/MediaDetail/Show/media_id/82437 |
| DPC 17947 | https://www.morphosource.org/Detail/MediaDetail/Show/media_id/82438 |
| DPC 20381 | https://www.morphosource.org/Detail/MediaDetail/Show/media_id/82488 |
| DPC 20965 | https://www.morphosource.org/Detail/MediaDetail/Show/media_id/82444 |
| DPC 21818 | https://www.morphosource.org/Detail/MediaDetail/Show/media_id/82440 |
